# Specific Anti-Leukemic Activity of the Peptide Warnericin RK and Analogues and Visualization of Their Effect on Cancer Cells by Chemical Raman Imaging

**DOI:** 10.1371/journal.pone.0162007

**Published:** 2016-09-06

**Authors:** Clémence Loiseau, Jacques Augenstreich, Adrienne Marchand, Etienne Harté, Martine Garcia, Julien Verdon, Marc Mesnil, Sophie Lecomte, Jean-Marc Berjeaud

**Affiliations:** 1 Laboratoire Ecologie & Biologie des Interactions, Equipe Microbiologie de l’Eau, Université de Poitiers, UMR CNRS 7267, Poitiers, France; 2 Institut de Chimie Biologie des Membranes et Nanoobjets, CNRS-Université de Bordeaux, UMR 5248, Pessac, France; 3 Laboratoire Inflammation, Tissus Epithéliaux & Cytokines-EA 4331, Poitiers, France; 4 Signalisation & Transports Ioniques Membranaires, CNRS ERL-7368, Poitiers, France; Shiraz University, ISLAMIC REPUBLIC OF IRAN

## Abstract

Antimicrobial peptides can be used as therapeutic agents against cancer cells. Warnericin RK and derivatives (WarnG20D and WarnF14V) were tested on various, solid tumor or leukemia, cancer cells. These peptides appeared to be cytotoxic on all the cell types tested, cancerous as well healthy, but very interestingly displayed no deleterious effect on healthy mononuclear cells. The mode of action of the peptide was proposed to be membranolytic, using chemical Raman imaging. Addition of peptide induced a large disorganization of the membrane leading to the loss of the content of inner compartments of Jurkat cell, whereas no effect was observed on the healthy mononuclear cells. The less hemolytic peptides WarnG20D and WarnF14V could be good candidates for the leukemia treatment.

## Introduction

Uncontrolled growth of cells, leading to cancer, constitutes a major cause of death worldwide. The Global Burden of Disease Cancer Collaboration have published that during 2013, cancer caused 8.2 million deaths and 14.9 million of new cancer were diagnosed worldwide [1]. The new therapies developed, during last decades, to be less harmful for the patients, as surgery and chemotherapy, were found to have, in too many cases, a relatively low successful rate and a high risk of reoccurrence [2]. The main problem with therapeutic molecules, including those from natural sources, concerns their insufficient selectivity and consequently their deleterious effects towards healthy cells [3, 4]. Moreover, cancerous cells develop frequently mechanisms of resistance and particularly through the pumping of anticancer drugs outside their cytosol [5].

As a consequence, anticancer peptides appear as promising candidates for cancer therapy. Indeed, these small molecules are expected to become efficient anticancer drugs because of their high selectivity for cancerous cells [6]. Moreover, most of these bioactive peptides interact directly with the cell membrane of the target cells, thus are supposed to induce less resistance mechanisms [6].

During the last two decades, a growing number of studies reported the cytotoxic activity against cancer cells of antimicrobial peptides (for reviews see [6–9]). In 2016, 198 peptides displaying anticancer or antitumor activity were listed in the Antimicrobial Peptide Database [10] available on http://aps.unmc.edu/AP/database/antiC.php. These multifunction antimicrobial peptides are used to fight against microbial invaders and constitute the first level of immune defense [11] that can be found in numerous eukaryotic organisms (plants, insects, reptiles, mammals,…) [12]. Anticancer peptides were divided in two classes [9]. The first group corresponds to peptides active against cancer cells while not being active against healthy mammalian cells, such as insect cecropins [13] and amphibian magainins [14, 15]. The second one corresponds to the cytotoxic molecules exerting the same activity towards healthy as well as cancerous cells. Bee venom mellitin [16], human neutrophil defensins [17, 18] and LL-37 [19] belong to this class with very low therapeutic potential.

There were only ten (from 198) of the antimicrobial peptides produced by bacteria which were described to display anticancer activities [10]. Firstly, microcinE492, a post-translational modified channel-forming bacteriocin produced by *Klebsiellapneumoniae*, was described to induce apoptosis in some cancer cell lines [20]. A hydrophobic analogue of Pep27, a *Streptococcus* signal peptide which was described to initiate the cell death program in *S*. *pneumonia* through signal transduction, was shown to induce apoptosis in various cancer cell lines [21]. Plantaricin A is a pheromone and antimicrobial linear peptide produced by *Lactobacillus plantarum* [22]. Its natural PlnA-22 analogue was shown to be toxic for cancerous GH_4_ cells but not for normal rat anterior pituitary cells [23]. More recently, baceridin, a new cyclic hexapeptide non ribosomal synthetized by a plant associated *Bacillus*, was described to induce apoptosis in tumor cells [24]. Three small lipopeptides, gageostatins A, B and C, produced by a marine *Bacillus subtilis*, were characterized and found cytotoxic for six human solid cancer cell lines [25]. In 2015, it was shown that two lasso peptides produced by *Streptomyces* species, sungsapin and chaxapeptin were both able to inhibit *in vitro* the cell invasion of human lung cancer cell line A549 [26, 27]. Finally, food preservative peptides nisin A and nisin Z were found to induce apoptosis of head and neck squamous cell carcinoma cells. Interestingly, nisin Z reduceedd tumorigenesis and extended survival of oral cancer floor-of-mouth mice [28].

The first anti-*Legionella* peptide, warnericin RK (WRK) was characterized in 2008 [29]. The anti-*Legionella* mode of action of WRK was described as detergent-like [30] and shown to be modulated by the lipid composition of the bacterial membrane [31]. Moreover, it was shown that WRK displayed a high hemolytic activity [29]. *Legionella* membrane is characterized by a high amount of phosphatidyl-choline (about 30%), which is known to be an eukaryotic phospholipid (present in red cell membranes for example), while only few bacterial species synthesize this phospholipid [32]. These data suggest that WRK could be cytotoxic for various mammalian cells, including malignant cells.

A collection of 12 anti-*Legionella* peptides produced by various species of *Staphylococci* were previously isolated and characterized [33]. These peptides were mostly already described for their hemolytic activity but were not known to be anti-*Legionella* [34, 35]. It was proposed, on the basis of their biological activities and mode of action, to separate these compounds in two classes [33]. The first class corresponds to peptides, resembling WRK, which are bactericidal and highly hemolytic. The peptides from the second group, with PSMα (Phenol-soluble modulin α) from *S*. *epidermidis* as an archetype, display a bacteriostatic mode of action and are poorly hemolytic [33, 36]. The latter peptide is a member of the PSM complex, composed of three peptides (PSMα, γ and δ), which was demonstrated to be implicated in the *S*. *epidermidis* biofilm detachment as well as displaying a pro-inflammatory activity [37–39]. Synthetic peptides were designed to display high anti-leukemic activity as WRK; but poor hemolytic effect as PSMα [40]. Two peptides were promising: WarnG20D in which the glycine residue in position 20 was replaced by an aspartic acid, and WarnF14V corresponding to the phenylalanine/valine substitution at position 14. Indeed, these peptides displayed an anti-*Legionella* activity close to the one of WRK; but were found less hemolytic than PSMα [40].

Confocal Raman spectroscopy is a non-invasive method to establish chemical cell imaging; it does not require chemical or biological markers. In 1990, Puppels *et al*. were the pioneers in introducing confocal Raman microscopy for studying single living cell [41]. Up to now, many studies have been done on different type of cells fixed or in solution. Confocal Raman microscopy combined with principal component analysis or clusters analysis allows also to localize compounds and compartments in cell [42, 43].

In this study, we showed that, the anti-*Legionella* bactericidal peptide, WRK displayed a cytotoxic activity towards cancerous cells from glioma, prostatic carcinoma and leukemia cell lines. However, the peptide was also found toxic for healthy glial and prostatic cells. Interestingly, the healthy mononuclear cells were unaffected by WRK. Low hemolytic variants of WRK, WarnG20D and WarnF14V also displayed anti-leukemia activity. Raman imaging allowed to point out the large damages induced by the peptides on the integrity of membranes of the leukemia cancerous cells (Jurkat), suggesting a membranolytic mode of action. In contrast, no modification was observed on healthy mononuclear cells in presence of the peptides.

## Materials and Methods

### Peptides

The peptides were purchased from GenScript (Piscataway, USA). Peptide concentration was determined by the bicinchoninic acid assay as described by the supplier (Sigma) with Bovine Serum Albumin as a standard. 25 μL of each sample were mixed with 200 μL of a solution of bicinchoninic acid and copper sulfate 50:1 (v/v). The preparation was incubated at 37°C for 30 minutes and the absorbance at 595 nm was measured by a microtiter plate reader OPSYS MR (ThermoLabsystems).

### Cells, culture media and growth conditions

Leukemia cell lines, Jurkat, K562 and KG1 were obtained from ATCC (American Type Culture Collection, VA, USA) and were cultured in RPMI 1640 (Roswell Park Memorial Institute), supplemented with fetal bovine serum (FBS) 10% for Jurkat and K562 cells and 20% for KG1 cells. The human prostate carcinoma cells, LNCap, were obtained from ATCC (American Type Culture Collection, VA, USA) and were cultured in DMEM (Dulbecco’s modified eagle medium, Invitrogen, CergyPontoise, France) with high glucose 4.5 g/L supplemented with 10% FBS (Lonza, Levallois-Perret, France), 100 IU/mL penicillin and 100 μg/mL streptomycin (Lonza). The adherent cancer cell lines rat glioma C6 cells are a generous gift of Pr. Naus (University of British Columbia) [44]. RWPE cells are immortalized prostatic cells and were obtained from ATCC. They were grown in keratinocyte serum free medium supplemented with 0,2 ng/ml epithelial growth factor and 30 μl/mL of bovine pituitary extract. Primary cultures of astrocytes were prepared from cortex and spinal cord of 1-day-old mice as previously described [45]. All experimental procedures involving animals were carried out in accordance with the guidelines of the French Agriculture and Forestry Ministry (decree 87849) and of the European Communities Council Directive (2010/63/UE). Mice were euthanized in accordance with the guidelines of the European Communities Council Directive (2010/63/UE). The study and protocol were approved by the local Committee on the ethics of animal experiments (ComEthEA–Region Poitou-Charentes) and does not need a particular permit. Since neonatal rodents are resistant to the hypoxia-inducing effects of CO_2_ and require longer exposure times to the agent, decapitation was used. Astrocytes were grown in the above-mentioned culture medium supplemented with glucose. They were purified by repetition of trypsinisation (0.25% trypsin and 0.02% EDTA, both from Sigma-Aldrich) and re-plating. The cells used in these experiments were obtained after the third passage. Human mononuclear cells (MNC) were isolated from fresh blood of 2 healthy individuals. The fresh whole blood was half diluted in RPMI 1640, supplemented with Ficoll (2:1 v/v) and centrifuged for 20 minutes at 1200*g* at 4°C. The cells from the interphase were extracted with a Pasteur pipette, transferred to RPMI 1640 medium supplemented with 10% FBS. Cells were washed three times, and then maintained in the medium at 4°C. All cells were cultured and maintained at 37°C in a humidified 5% CO_2_ incubator.

### Hemolytic activity assays

Hemolytic activity of the peptides was determined by measuring the released hemoglobin from human erythrocytes. Prior to the assay, the fresh human blood was centrifuged (2000 *g*, 3 min, 4°C) to collect red blood cells. Erythrocytes were washed in phosphate buffered saline (PBS) until the supernatant was clear (5 times). Reactions were performed in 1 mL mixtures containing 1% erythrocytes (v/v) and variable amount of peptides or an equivalent volume of PBS buffer. Serial two-fold dilutions of peptides were performed in PBS buffer and added to 1% human erythrocytes at a starting concentration of 50 μg/mL. The reaction mixtures were incubated at 37°C for 30 min. Erythrocytes were removed by centrifugation (2000 g, 3 min, at 4°C) and the A_576nm_ of the supernatant was determined with a spectrophotometer ThermoSpectronicBiomate 3 (Avantec). 100% hemolysis was measured by adding 0.1% Triton X-100 to the reaction mixture instead of peptides solution.

Human blood was obtained from the French national blood transfusion organization, "Etablissement Français du Sang—Centre Atlantique", under the agreement number CA-PLER-2014 089. Donors gave their written and informed consent for the use of blood samples in research.

### Membrane permeabilization assays

Cells were washed and resuspended in phosphate buffer saline (PBS) at a concentration of 10^6^ cells /mL. The cells suspension was treated with various concentrations (25, 12.5, 6, 3, 1 and 0 μg/mL), of peptides during 45 min at 37°C. The cells were then treated during 15 min with 5 μl of propidium iodide (PI), a fluorescent dye which only penetrates the cells with damaged cell membrane, before flow cytometry analysis. Flow cytometric measurements were performed on a FACSConto II flow cytometer (BD Bosciences) with a 488 nm argon excitation laser. A total of 100 000 events were analyzed in each sample, using BD FACSDiVa 6 software (BD Biosciences) for data acquisition and analysis. LD_50_ were calculated from the linear regression of the rate of permeabilized cells as a function of the peptide concentration expressed in μM. 100% permeabilization was measured by adding 0.1% Triton X-100 to the reaction mixture instead of peptides solution.

### Confocal Raman microscopy

To achieve the Confocal Raman spectroscopy, Jurkat and MNC cells, the cells were centrifuged at 1000*g* for 3 minutes to replace the RPMI medium and diluted to reach 3.10^5^ cells/mL in phosphate buffer saline (PBS) at pH 7.4. The cells were transferred in a Petri dish with a glass microscope slide at the bottom to avoid the signal of the plastic from the dish. The peptides WRK, WarnG20D, WarnF14V and PSMα were added at 10μM on cell suspensions for 1 hour. Raman spectra were recorded on single cell before the peptide addition, and one hour after the addition of the peptide at room temperature.

Raman spectra were recorded using a WITec (Ulm, Germany) Alpha300RS confocal Raman microscope. The excitation wavelength of 532 nm was provided by a frequency-doubled Nd:YAG laser. The beam was focused on the sample using a water immersion Olympus objective (63X/1 NA). The power at the sample was close to 10mW. The sample was located on a piezoelectrically driven microscope scanning stage. The spatial resolution for the Raman scattering is 300 nm for x and y and 500 nm for z due to the diffraction limit. The integration time for each spectrum was 50 ms. The measure was performed on an area of 20 μm x 20 μm with 100 lines and 100 points by line. Thus the time of a single cell scan was about 10 minutes and allowed the cells to stay intact. The spectra were collected and analyzed using the WITec Control and WITec Project Manager softwares (Ulm, Germany). The Hierarchical Cluster Analysis (HCA) was used to identify regions of the sample that have similar spectral response by clustering the spectra into groups or clusters such that differences in the intracluster spectral responses are minimized while simultaneously maximizing the intercluster differences between spectral responses [46, 47].

## Results and Discussion

### Anticancer activity of WRK

**Fig 1:**
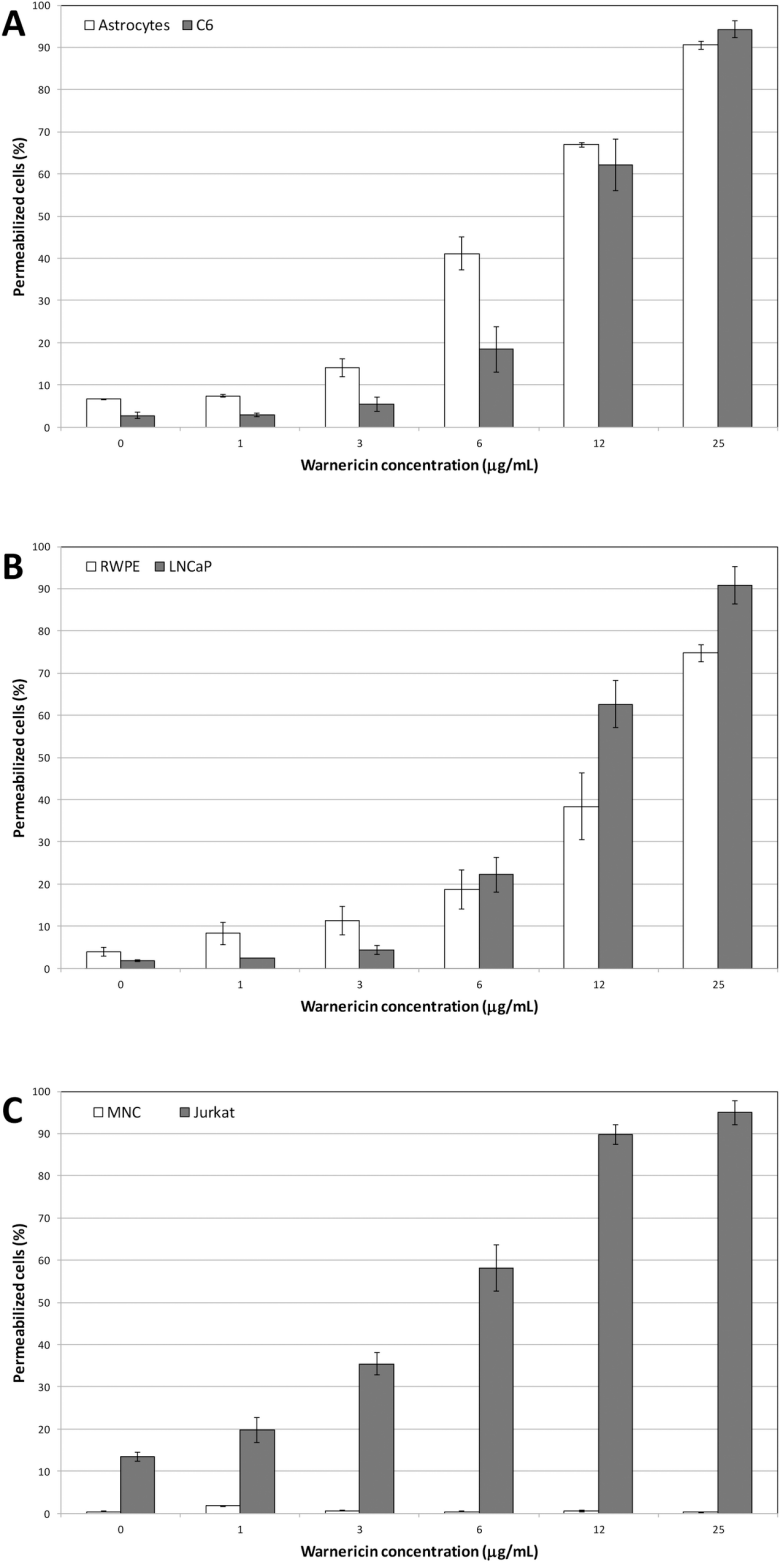
Effect of various concentrations of WRK on **A**: glial cancerous (C6), and healthy (Astrocytes) cells; **B**: prostatic cancerous (LNCap) and healthy (RWPE) cells; **C**: peripheral mononuclear cancerous (Jurkat) and healthy (MNC) cells. Results are expressed as the percentage of cell permeabilization in comparison to the negative (PBS buffer) and positive (0.1% Triton X-100) controls. Experiments were performed in triplicates, and the results are expressed as means. Bar scales indicate standard deviation.

In order to detect the anticancer activity of WRK, various concentrations of the peptide were tested on suspensions of different cancer cell lines as following: one derived from prostatic tumor (LNCaP), one glioma cell line (C6) and the Jurkat leukemia cell line. The cytotoxicity of the peptide was followed by flow cytometry using propidium iodide (PI) staining which penetrates the cells with degraded cytoplasmic membrane. In parallel, same experiments were conducted on the corresponding healthy prostatic cells (RWPE), glial cells (Astrocytes) and peripheral blood mononuclear cells (MNC). Fig 1 presents the rate of permeabilized cancerous and healthy tested cells. All the cancerous cells appeared sensitive to low concentration of the peptide since a 6 μg/mL dose induced about 20% of permeabilization for the solid cancer cells (C6 and LNCaP, Fig 1A and 1B) and near 60% for the leukemia cells (Jurkat, Fig 1C). Moreover, addition of peptide at 25 μg/mL induced the permeabilization of more than 90% of all the cells. Table 1 listed the median lethal dose (LD_50_) for each cell lines. The high toxicity of WRK towards all the tested cancer cells, from solid tumor as leukemia was confirmed. Jurkat cells seem about twice sensitive (LD_50_ = 2.11 μM) to the peptide than the other cancer cells (LD_50_ = 4.14 and 3.98 μM for C6 and LNCaP respectively). Considering the healthy cells WRK was found toxic for prostatic and glial cells (Fig 1). WRK applied at 25μg/mL induced more than 70% and 90% of permeabilization for RWPE cells (Fig 1B) and astrocytes (Fig 1A), respectively. On the contrary, the MNC were found completely insensitive to WRK. The percentage of permeabilized cells were identical for cells treated by 25 μg/mL of WRK or for untreated cells (Fig 1C). The LD_50_ for these healthy cells were of the same order as the cancerous cells (Table 1) except for MNC for which appeared totally insensitive to WRK at the tested doses.

**Table 1:** Median lethal dose (LD_50_) of WRK on cancer cell lines tested.

	Glial cells		Prostatic cells		Leukocytes			
Cell line	C6	Astrocytes	LNCaP	RWPE	Jurkat	KG1	K562	MNC
LD_50_ (μM)	4.14	3.36	3.98	6.37	2.11	2.18	4.22	ND

PSMα is an other anti-*Legionella* peptide produced by *S*. *epidermidis* which displayed an MIC similar to the WRK one. However, it differs from WRK because of its bacteriostatic mode of action and its hemolytic activity which is about three times lower than WRK [30]. Thus, PSMα was also assayed against the same three types of cancerous cells and corresponding healthy cells (see supplementary information S1 Fig). Interestingly, the rate of permeabilized cells treated by PSMα was found very low whatever the cells, indicating that PSMα displayed no toxic activity on healthy or cancer cells.

**Fig 2:**
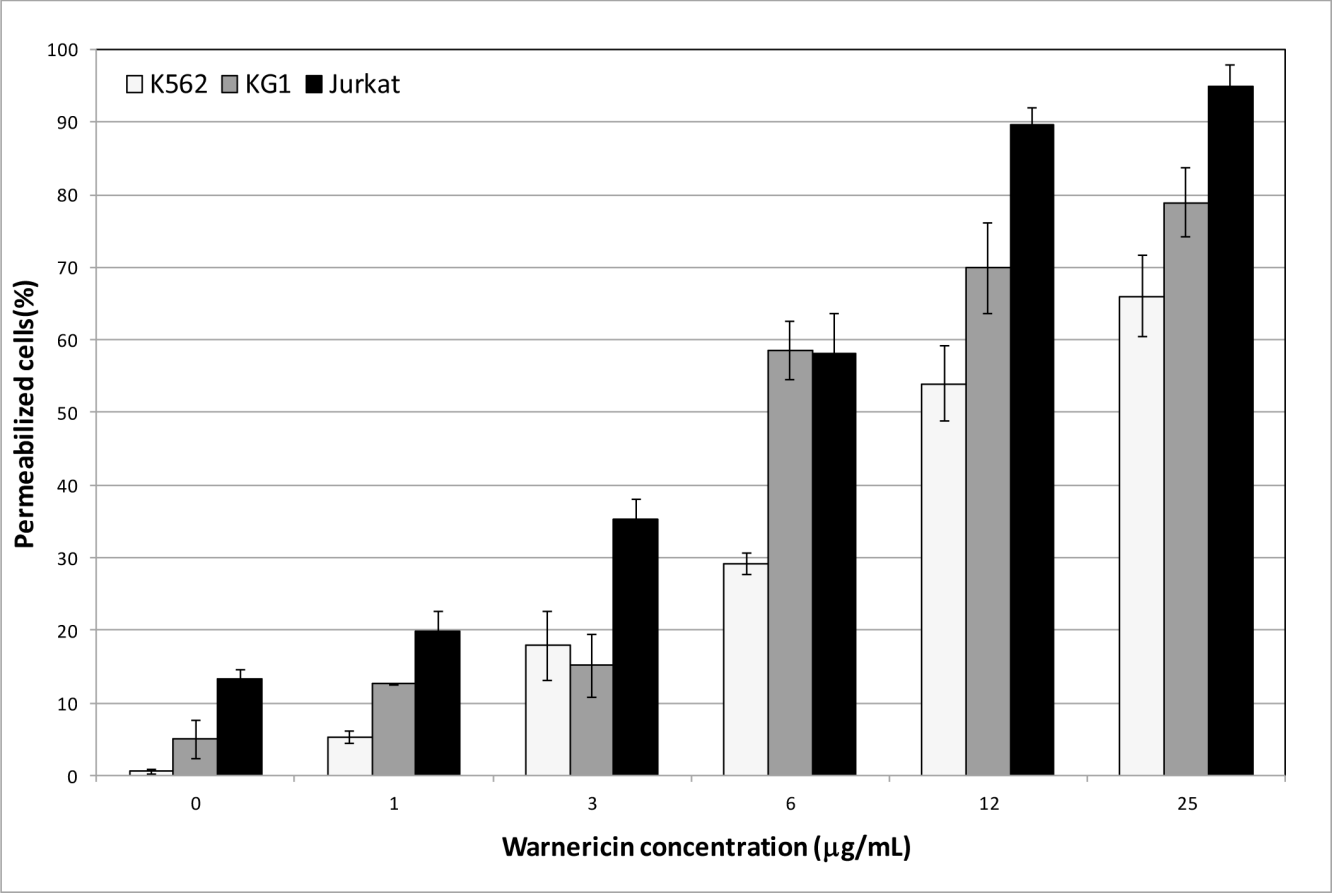
Effect of various concentrations of WRK on leukemia cell lines Jurkat, KG1 and K562. Results are expressed as the percentage of cell permeabilization in comparison to the negative (PBS buffer) and positive (0.1% Triton X-100) controls. Experiments were performed in triplicates, and the results are expressed as means. Bar scales indicate standard deviations.

The non-toxicity of WRK for MNC indicates that this peptide could be a good candidate for leukemia therapy. However, Jurkat are T-lymphocytes which originated from an acute T-cell leukemia. We tested the WRK toxicity on two other different leukocytes cell lines KG1 and K562. Both are macrophages cell types, corresponding to an acute myelogenous leukemia and a chronic myelogenous leukemia respectively. Indeed, the bacterial anticancer peptide, microcin E492, was found to be cytotoxic for Jurkat cells but inactive against KG1 cells [20]. The rates of permeabilized leukemic cells by WRK are presented in Fig 2. Even if the Jurkat cells appeared the most sensitive to the peptide, with 95% of permeabilized cells at 25 μg/mL, compared to about 80% and 65% for KG1 and K562 cells respectively, all the leukemia cell lines were sensitive, in a dose-response manner to WRK. KG1 and K562 cells were found insensitive to PSMα (data not shown).

Taken together, these results suggest that the mammalian cells toxicity of WRK could be related to its bactericidal mode of action which was demonstrated to be membranolytic (detergent-like) and dependent on the membrane lipids of *Legionella pneumophila* [30, 35], whereas the non-cytotoxic PSMα, is bacteriostatic.

This study clearly demonstrates that WRK has strong activity against cancer cells. It has to be classified in the second group of anticancer peptides, cytotoxic for both healthy and cancerous cells, for prostatic and glial cells. But more interestingly, WRK has to be classified in the first class, active towards leukemia cancer cells but not healthy cells (MNC), that may be very promising for leukemia anticancer therapy. Nevertheless, WRK was described to be highly hemolytic [29], which disqualifies it for possible anticancer therapy application.

### Anti-leukemia activity of low hemolytic WRK variants

**Fig 3:**
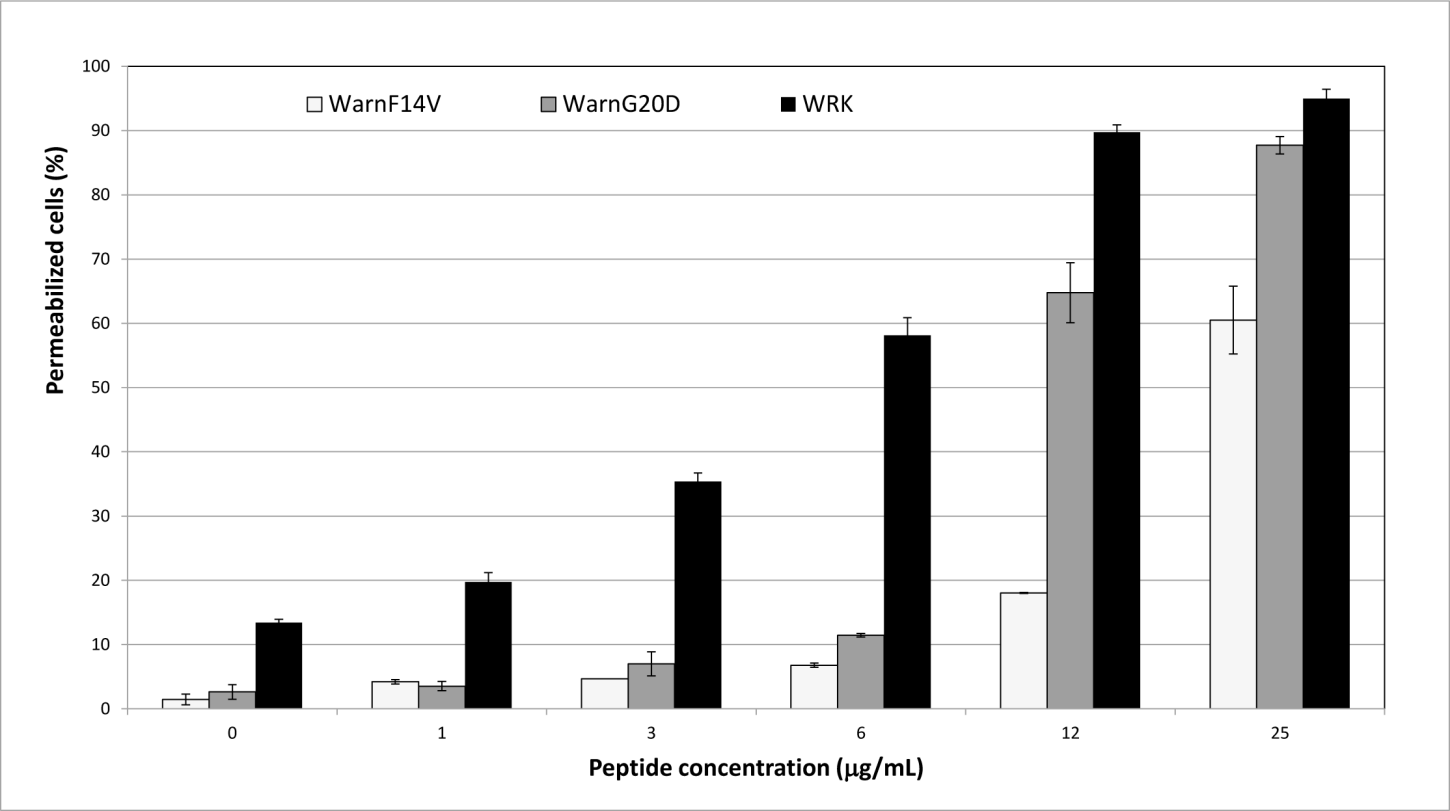
Effect of various concentrations of WRK, Warn G20D and WarnF14V on Jurkat cells. Results are expressed as the percentage of cell permeabilization in comparison to the negative (PBS buffer) and positive (0.1% Triton X-100) controls. Experiments were performed in triplicates, and the results are expressed as means. Bar scales indicate standard deviations.

In a previous study [40], we designed and selected synthetic variants of WRK which were as active as WRK against Legionella with a decreased hemolytic activity. The most interesting variants, WarnG20D and WarnF14V, were tested for their anti-leukemia against Jurkat cells. The results, obtained by flow cytometry, are presented in Fig 3. Even if WRK (black bars) remains the most active peptide against Jurkat cells, WarnF14V (white bars) and WarnG20D (grey bars) displayed also cytotoxicity. WarnG20D and WarnF14V induced, at 25 μg/mL, rates of 88% and 60% of permeabilized cells, respectively. The deduced LD_50_ for these peptides (Table 2), 5.39 and 8.79 μM for WarnG20D and WarnF14V respectively, were found higher than the one of WRK (2.11 μM) but were inferior to 10 μM. Considering their hemolytic activity, the minimal concentration responsible for the lysis of 10% of red blood cells was around 12.5 μM for both variants, whereas it was measured at 2.78 μM for WRK (Table 2). An anti-leukemia index, analog to the therapeutic index, was calculated by divided the 10% hemolysis concentration by the LD_50_ for each peptide (Table 2). The highest value (2.293) was obtained for WarnG20D, by comparison with those of WarnF14V and WRK (1.422 and 1.317 respectively). Moreover, both WRK variants were found inactive against MNC cells (data not shown), which highlights the potential of WarnG20D for leukemia therapy.

**Table 2:** Primary sequences and biological activities of WRK and analogs WarnG20D and WarnF14V.

Name	Sequence	LD_50_^a^ (μM)	10% Hemolysis concentration^b^ (μM)	Anti-leukemia index^c^
WRK	MQFITDLIKKAVDFFKGLFGNK	2.11	2.78	1.317
WarnG20D	MQFITDLIKKAVDFFKGLFDNK	5.39	12.36	2.293
WarnF14V	MQFITDLIKKAVDVFKGLFGNK	8.79	12.5	1.422

^a^ Cytotoxic activity was determined on Jurkat cells.

^b^ 10% Hemolysis concentration corresponds to the peptide concentration which induces the lysis of 10% of the red cells in the sample.

^c^ Anti-leukemia index corresponds to the ratio: 10% Hemolysis concentration / LD_50_.

### Mode of action of WRK and low hemolytic variants on Jurkat cells by confocal Raman microscopy and imaging

Jurkat cells incubated with WRK, with low hemolytic variants (WarnG20D and WarnF14V) or PSMα were analyzed by microscopy and confocal Raman microspectroscopy. The effect of each peptide, before and 1 hour after peptide addition at 10 μg/mL, on the Jurkat cell population was firstly estimated by optical microscopy (data not shown). WRK was found to induce lysis of the Jurkat cells. Indeed, about 50% of the cells were still observable after 1 hour. A small decrease (less than 10%) in the Jurkat cell population was observed in presence of WarnG20D and WarnF14V. In contrast, PSMα had no effect on the Jurkat population, which is in agreement with the results presented above. It has to be noticed that the cells are not fixed thus they floated in the buffer; the population count under the microscope objective was only an estimation. These results are in good agreement with the higher permeabilization of Jurkat cell by WRK than variants presented in Fig 3. Indeed, at 6 μg/mL, about 55% of cells were permeabilized by WRK but only 6 to 10% by WarnF14V and WarnG20D respectively.

**Fig 4:**
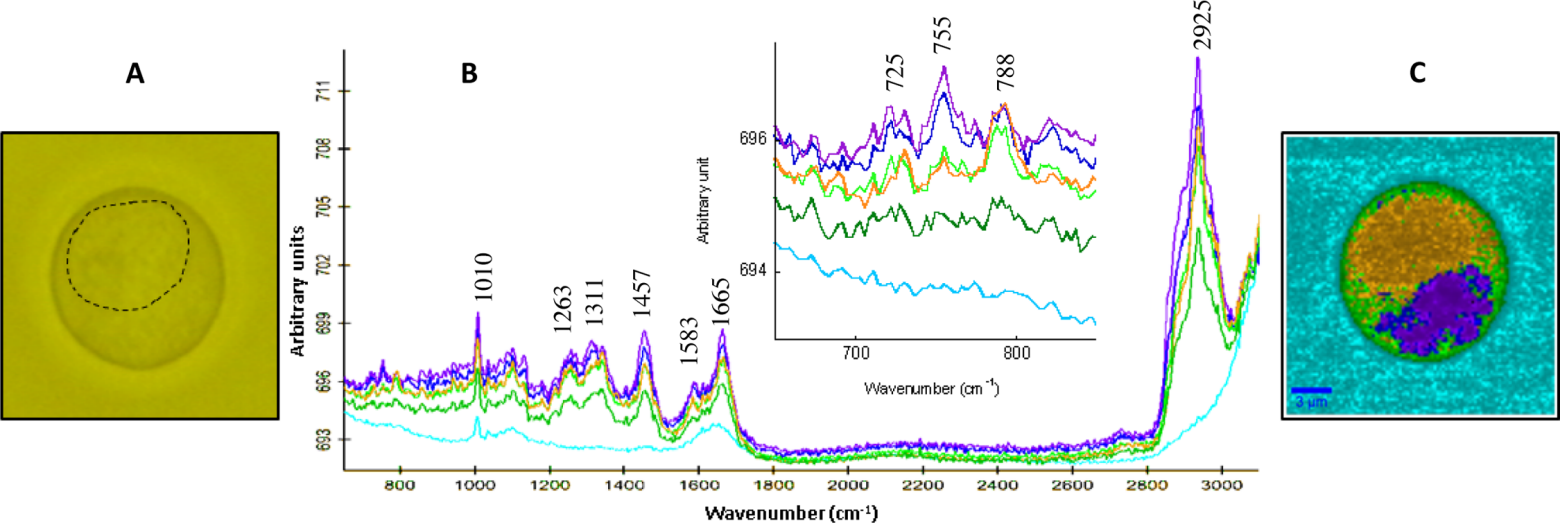
**(A)** Light microscopy image of Jurkat cell. The straights line reveals the contrast due to the nucleus. **(B)** Cluster average spectra from HCA results. **(C)** Chemical image of Jurkat cell building from the HCA results. The colors correspond to the Raman spectra generated by HCA methods (see B) and characteristic of the different cell compartments (see description in the text). Arrows allow to highlight the differences between cells treated and untreated by the peptides.

Confocal Raman spectroscopy on unique cell was performed, to image the different compartments of the cell before peptide addition. Fig 4A shows the optical image of the Jurkat cell, the contrast observed in the middle of cell reveals the presence of the nucleus. Raman spectra were recorded on the same zone and hierarchical cluster analysis (HCA) process was performed with six cluster areas in the mapping (Fig 4B). Shie et al. [41] studied the effect of doxorubicin on Jurkat cell by Confocal Raman spectroscopy. The Raman spectra of both Jurkat cells (before treatment) reported by Shie et al. or in our study are very similar. The fingerprints of the classical molecules present in cells, as lipids, proteins and nucleic acids were thus detected. The main bands are labeled on the Raman spectra (Fig 4B). The band assignments are listed in Table 3.

**Table 3:** Raman band assignments according to references [47–49].

Band (cm^-1^)	Assignment
725	DNA/RNA (adenine ring breathing)
755	Proteins (tryptophan symmetric ring breathing)
788	DNA/RNA (thymine, uracil, cytosine ring breathing) and DNA backbone (O-P-O stretching)
1010	Proteins (phenylalanine symmetric ring breathing)
1100	Lipids, Proteins (C-C stretching)
1137	Lipids, Proteins (C-C stretching)
1263	Proteins (Amide III)
1311	Lipids, Proteins, DNA/RNA
1348	Lipids, Proteins, DNA/RNA
1457	Lipids (CH_2_ CH_3_ binding)
1583	DNA/RNA (guanine, adenine)
1665	Proteins (Amide I)
2850–2950	Lipids, proteins (CH_2_, CH_3_, symmetric and antisymmetric stretching and CH stretching)
3080	Lipids (C = C-H, CH stretching)

Cell chemical imaging was reconstructed using the HCA process classification (Fig 4C). The different colors represent the average of all identical spectra recorded in probed area. The cyan domain (corresponding to cyan spectrum) shows mainly the signature of water with strong band at 3300 cm^-1^ and small band at 1640 cm^-1^ assigned to νOH and δOH, respectively.

A thin band is observed at 1003 cm^-1^; it seems to be a signature of phenylalanine amino acid. It seems to be a residual component of the cell culture medium. The other spectra exhibit signature of lipids, and proteins. The range above 2800 cm^-1^ is dominated by CH stretching vibrations of lipids and proteins. Proteins are revealed by the characteristic amide I and amide III bands at 1665 and 1263 cm^-1^ respectively. HCA analysis points out differences in the low wavenumber range, between 800 and 650 cm^-1^ (see zoom in Fig 4B). This domain is characteristic of the DNA or RNA bases vibration modes and of particular amino acid residue (tryptophan) (Table 3) [49–51]. The two green domains correspond to the localization of the membrane and the cytoplasm. The lipid and protein contributions are small related to weak bands observed and the contribution of water increases, as expected. The orange cluster is specific spectrum of the nucleus. The chemical image of the nucleus is well correlated to the optical image (dashed line Fig 4A). The additional band at 788 cm^-1^ is associated with Thymine, Uracil and Cytosine ring breathing modes of DNA and RNA bases, and also contains contributions from O-P-O DNA backbone. The blue and purple spectra are similar to the other in the high wavenumber range. The difference with the other compartments is mainly due to the enhancement of the band at 755 cm^-1^ assigned to tryptophan symmetric ring breathing mode. This indicates an increase in the protein content. A weak increase of the amide I and amide III bands is also observed. Lipids contribution is also more important in this range, as revealed by the high intensity of the CH_2_ and CH_3_ stretching modes between 2800 and 3000 cm^-1^. This domain close to the nuclei could be assigned to the endoplasmic reticulum, where takes place the lipid metabolism and protein synthesis.

**Fig 5:**
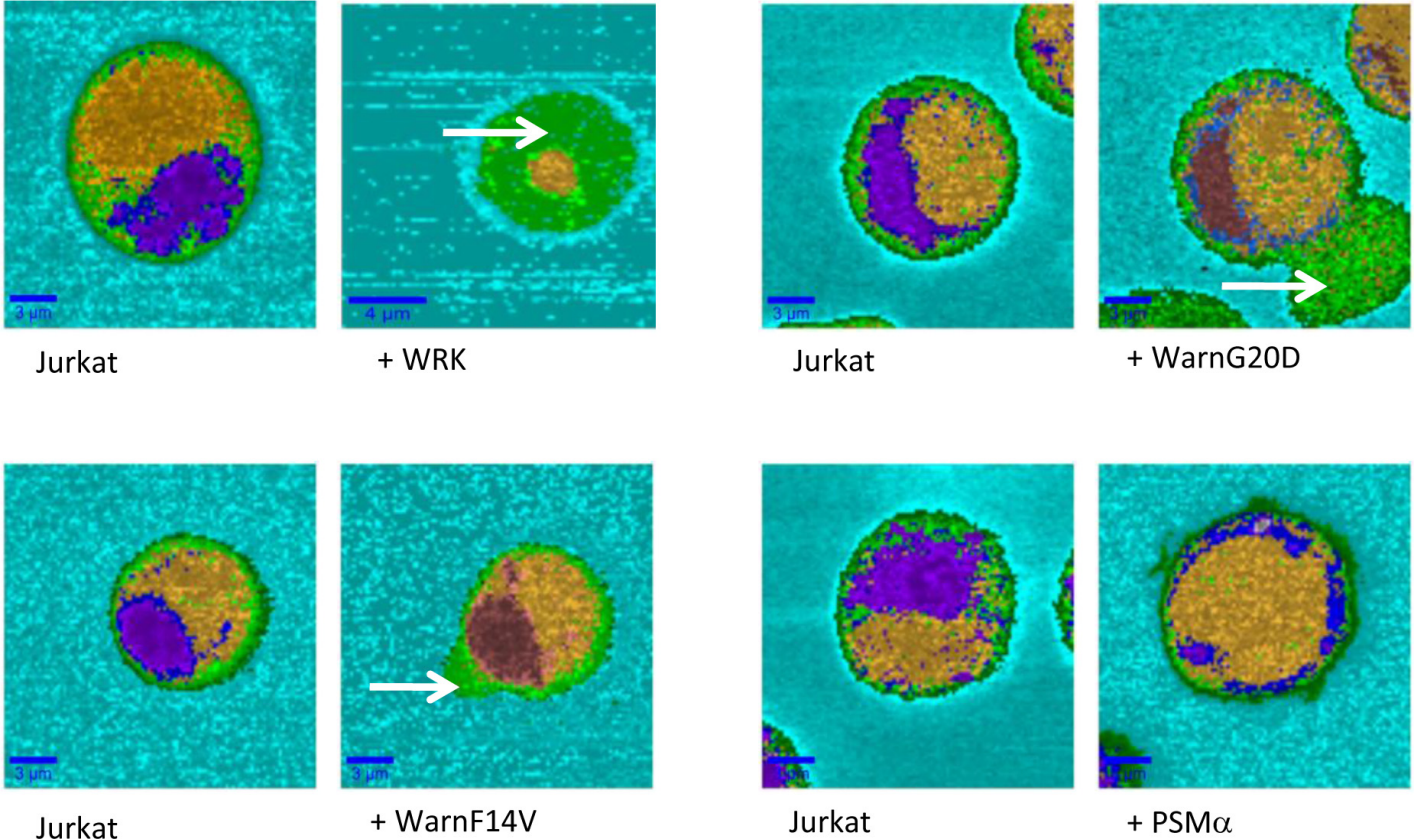
Chemical images of Jurkat cell before the peptide injection noted (Jurkat) and 1 hour after injection of the different peptides (notified by + peptide’s name). Scale bars are notified in each image. The colors correspond to the Raman spectra generated by HCA methods and characteristic of the different cell compartments.

Fig 5 shows chemical images of the Jurkat cell before and 1 hour after injection of peptides. For all analyzed cells the same chemical images were obtained. The different compartments are still represented in orange for the nucleus, blue and purple for endoplasmic reticulum and green for membrane and cytoplasm. Addition of WRK created large Jurkat cell damages and the image of the cell still present in the buffer was modified (Fig 5). We repeated the observation for at least three remaining cells in the medium. The recorded spectra are only characteristic of cytoplasm. The arrow Fig 5 pointed out the loss of inner compartments of the cell. The nucleus and the endoplasmic reticulum were both not detected, leading to conclude that WRK is an effective membrane active peptide, as demonstrated for its activity on *Legionnella* [30]. Derivative peptides lead to less drastic effect on cells. Deformations of cells were mainly observed after addition of WarnG20D and WarnF14V. The nucleus and endoplasmic reticulum compartment are still observed. Small modification of the relative intensity of the band in the low wavenumber range (725, 755, 785 cm^-1^) are observed, leading to the modification of the color on the chemical images for the reticulum endoplasmic domain (Fig 5). However, the outline of the compartments is still observable. The deformed areas are related to the cytoplasm and cell membrane domain and could correspond to intracellular material leakage (see white arrow in Fig 5 for both peptides WarnG20D and WarnF14V). PSMα injection has no effect on Jurkat cell morphology, in agreement with results described previously. The compartment integrity was still observed with no cell deformation (Fig 5). Finally, WRK is the most active peptide on Jurkat cells allowing to large destruction of cell. The other active peptides (WarnG20D and WarnF14V) induce first deformation of the cell that can lead after longer time to the cell lysis, explaining their toxicity.

**Fig 6:**
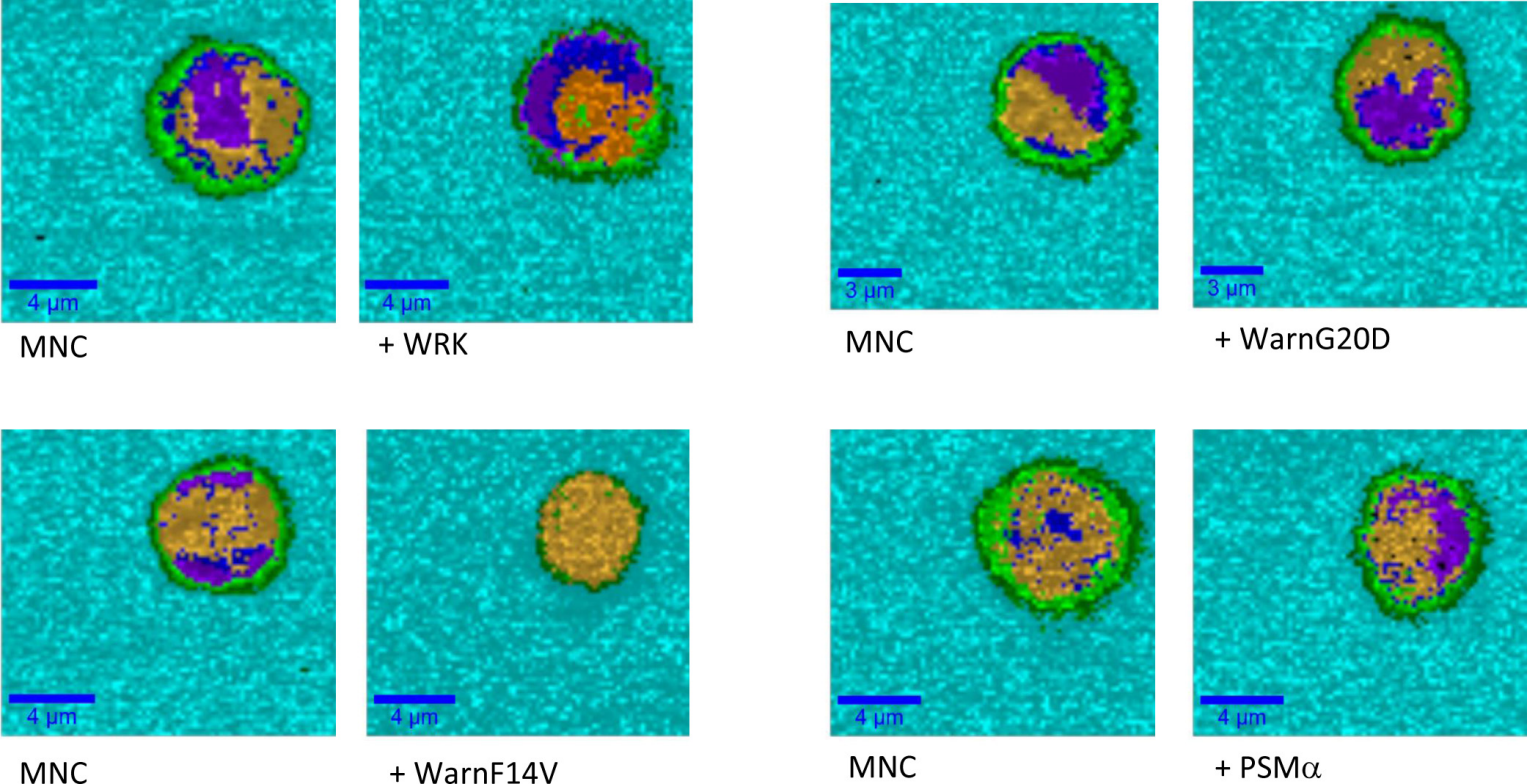
Chemical images of MNC cells before the peptide injection noted (MNC) and 1 hour after injection of the different peptides (notified by + peptide name). Scale bars are notified in each image. The colors correspond to the Raman spectra generated by HCA methods and characteristic of the different cell compartments.

The peptides were also added in the medium containing the healthy MNC cells to evaluate their selectivity against cancerous cells. The MNC cells are smaller than Jurkat cells. After addition of the peptides the cell number remains similar even after one hour following the peptide injection (data not shown). Fig 6 shows the chemical image of a unique MNC cell before and 1 hour after peptide addition. Similar Raman spectra were observed with similar peaks assigned to lipids, proteins and nucleic acids, as described for Jurkat cells (Fig 4 and Table 3). Whatever the peptide the integrity of the MNC cells is conserved. The difference in the images can be due to orientation of the cell under the laser beam. Indeed, the cell is floating in the medium, if it rotates the repartition of the various compartments can be modified. But from these images we can conclude that these peptides, added at similar concentration (10 μg/mL), have no activity on the healthy cells (MNC) in contrast to the results obtained on leukemic cells.

### Conclusions

WRK is cytotoxic for all the tested cells, leukemic cells (Jurkat, KG1, KS62) prostatic and glial cancer cells, prostatic healthy cells and astrocytes, but not for healthy mononuclear cells (MNC).

Low hemolytic WRK derivative peptides, WarnG20D or WarnF14V, were also found to be active against Jurkat cells but not toxic for MNC. Their mode of action was studied using chemical Raman imaging without any labeling of cells. The membranes of the cancer cells, treated by the peptides, are strongly affected, the cell compartments were disorganized and Raman signature of cytoplasm was only observed. This is in agreement with the postulated detergent like mechanism of action of WRK on *Legionella* [27]. At similar peptides concentration, no modification of the MNC cells was observed. Low hemolytic WRK variant, WarnG20D, according to its anti-leukemia activity could be good candidates for treatment of leukemia. However, because of the putative cytotoxicity of the peptide for cells other than MNC, it would be interesting to test the efficacy for leukemia therapy of WarnG20D immobilized in an extracorporeal shunt system as proposed by Qiao et al. [52] for the anticancer enzyme L-Asnase.

## Supporting Information

S1 Fig Effect of various concentrations of PSMα on glial cancerous (C6), and healthy (Astrocytes) cells; prostatic cancerous (LNCap) and healthy (RWPE) cells; peripheral mononuclear cancerous (Jurkat) and healthy (MNC) cells. (TIF)
