# Supplementary figures and images for: Specific Anti-Leukemic Activity of the Peptide Warnericin RK and Analogues and Visualization of Their Effect on Cancer Cells by Chemical Raman Imaging
Source: PLoS One. 2016 Sep 6;11(9):e0162007. doi: 10.1371/journal.pone.0162007 (PMC5012605; doi:10.1371/journal.pone.0162007)

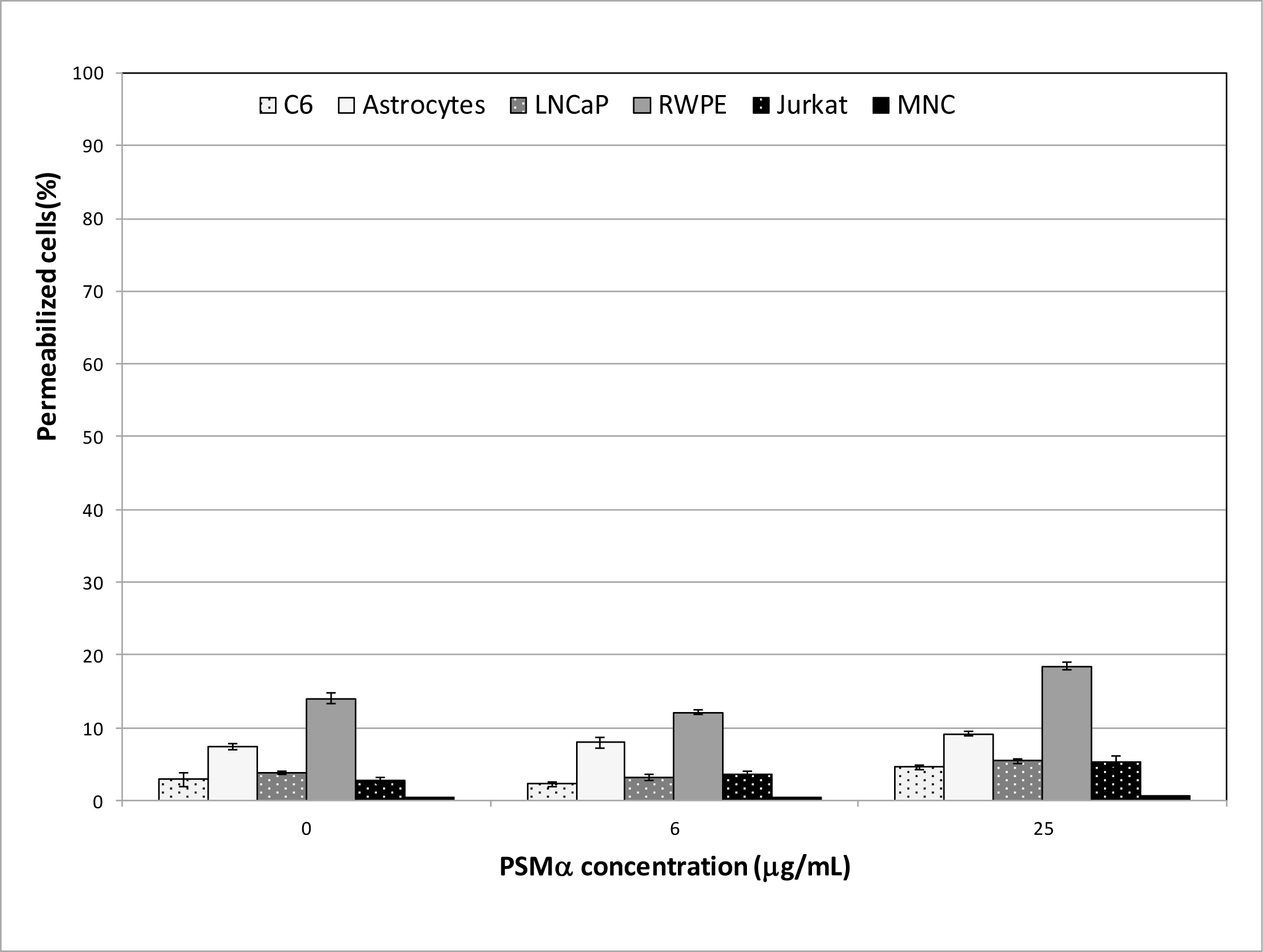

Supplement: S1 Fig — (TIF) [file pone.0162007.s001.tif]
